# Encapsulation of Vecuronium and Rocuronium by Sugammadex Investigated by Surface-Enhanced Raman Spectroscopy

**DOI:** 10.3390/molecules30020231

**Published:** 2025-01-09

**Authors:** Adriana Kenđel, Ivo Piantanida, Snežana Miljanić

**Affiliations:** 1 Department of Chemistry, Faculty of Science, University of Zagreb, Horvatovac 102a, 10000 Zagreb, Croatia; akendel@chem.pmf.hr; 2 Institute Ruđer Bošković, Division of Organic Chemistry & Biochemistry, Bijenička Cesta 54, 10000 Zagreb, Croatia; ivo.piantanida@irb.hr

**Keywords:** vecuronium, rocuronium, γ-cyclodextrin, sugammadex, SERS

## Abstract

Aiming toward a novel, noninvasive technique, with a real-time potential application in the monitoring of the complexation of steroidal neuromuscular blocker drugs Vecuronium (**Vec**) and Rocuronium (**Roc**) with sugammadex (**SDX**, medication for the reversal of neuromuscular blockade induced by **Vec** or **Roc** in general anesthesia), we developed proof-of-principle methodology based on surface-enhanced Raman spectroscopy (SERS). Silver nanoparticles prepared by the reduction of silver ions with hydroxylamine hydrochloride were used as SERS-active substrates, additionally aggregated with calcium nitrate as needed. The **Vec** and **Roc** SERS spectra were obtained within the biorelevant 5 × 10^−7^–1 × 10^−4^ M range, as well as the SERS of **SDX**, though the latter was observed only in the presence of the aggregating agent. **SDX**/drug complexes at a 1/1 molar ratio revealed significant spectral changes in the vibrational bands of the **SDX** glucose rings and the drug steroid rings, implying that the insertion of **Vec** and **Roc** molecules into the **SDX** cavity was not only driven by attractive electrostatic interactions between the positively charged cyclic unit of the drug and the negative carboxylate groups of cyclodextrin but also supported by hydrophobic interactions between the host cyclodextrin and the guest drug molecule. The observed changes in SERS signals are applicable in biorelevant conditions and support further studies of **SDX**/drug complexes in vivo.

## 1. Introduction

**Figure 1:**
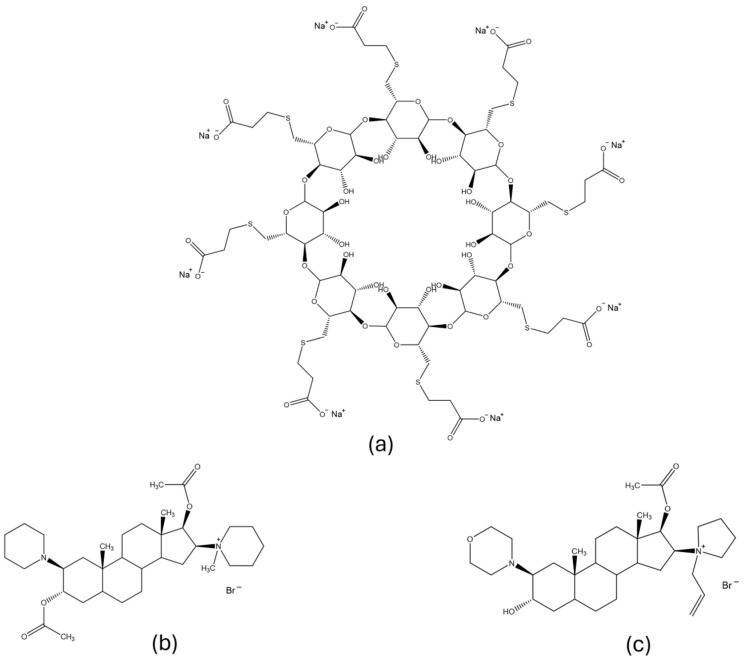
Molecular structures of (**a**) sugammadex, (**b**) Vecuronium bromide, and (**c**) Rocuronium bromide.

Neuromuscular blocking agents are routinely used in anesthesia to achieve muscle relaxation during surgery and prevent interference with voluntary or reflex muscle movements [1,2]. They block acetylcholine-induced neurotransmission by binding to the nicotinic acetylcholine receptor at the neuromuscular junction [3]. Neuromuscular blockers are divided into two groups: depolarizing and non-depolarizing agents. Depolarizing drugs act as agonists, like acetylcholine at nicotinic receptors, while non-depolarizing drugs compete with acetylcholine for nicotinic receptor binding sites and prevent neurotransmitter–receptor binding. Non-depolarizing agents are either benzylisoquinoliniums or aminosteroids. Examples of very common and extensively used aminosteroid-based non-depolarizing neuromuscular blocking drugs are Rocuronium (**Roc**) and Vecuronium (**Vec**) (Figure 1) [2,3,4,5].

After surgery, there are two ways to restore neuromuscular transmission when non-depolarizing blocking agents are used [6]. One way is the use of cholinesterase inhibitors such as neostigmine [7], while the other is chemical encapsulation of the blocking agent with host molecules [6,8]. The main disadvantages of using inhibitors are the frequent occurrence of side effects and inability to restore deep paralysis [6]. These difficulties do not arise when an encapsulating reagent is used. For Vecuronium bromide and Rocuronium bromide, the binding host is a compound named sugammadex (Figure 1, **SDX**), which forms a complex with steroid drug molecules in a 1/1 molar ratio [2,9]. Sugammadex is a modified γ-cyclodextrin, a cyclic oligosaccharide composed of eight sugar moieties that form a lipophilic core, additionally extended by eight carboxyl thioether substituents, thus enabling the encapsulation of smaller lipophilic molecules [5]. By complexing the drug, sugammadex rapidly reverses neuromuscular blockade caused by Vecuronium or Rocuronium. The binding of free drug molecules with sugammadex in plasma leads to a decrease in drug concentration, followed by a concentration gradient between the drug molecules in the plasma and the neuromuscular junction, which further on causes the drug molecules to return to the plasma and be encapsulated by sugammadex [10,11].

Various techniques have been used to determine drugs in cell fluids and to study sugammadex/drug interactions. Hence, calorimetry [9], spectroscopy [12], chromatography [13], together with NMR spectrometry [14], and X-ray diffraction [15] were used for the determination of Vecuronium, whereas calorimetry [9] and chromatography alone [16,17] and in tandem with mass spectrometry [18,19] were applied to detect Rocuronium. **SDX**/drug complexes were experimentally studied using isothermal titration calorimetry [9], X-ray diffraction [2], and the NMR technique [20] and predicted using extensive molecular modeling [8]. However, to our knowledge, no method has been applied for the structural characterization of **SDX**/drug complexes in aqueous solution. In order to gain insight into the structure-related behavior of the **SDX**/drug complexes in an environment similar to cellular conditions, we propose the use of a vibrational technique that enables the structural characterization of compounds in an aqueous medium at concentrations lower than micromolar, avoiding deuterated solvents or additional marker molecules in the system. As a first step in the development of sensors for amino steroid drugs and their complexes in biological fluids, it is of great importance to find a method that will provide structural information about complex sugammadex (**SDX**)/drugs while using only water as a solvent.

Surface-enhanced Raman scattering (SERS) spectroscopy is a highly sensitive vibrational, spectroscopic technique used for the structural characterization of molecules located near the rough surface of a metal substrate, called the SERS substrate [21]. Due to the laser radiation-induced excitation of surface plasmons on SERS substrates and chemical binding of analyte molecules to the enhancing metal surface, Raman scattering can be enhanced up to 10^10^ times if compared to normal Raman spectroscopy [22]. Therefore, it is possible to detect analytes at submicromolar concentrations in aqueous solutions, when the appropriate measurement conditions for the SERS experiment are applied and the optimal metal substrate is used.

The complexation of drugs by sugammadex (**SDX**) should occur rapidly and effectively. Real-time monitoring may be critical for patient care, making it important to use a noninvasive and straightforward technique for the in situ monitoring of patient blood. The sensitivity of Raman spectroscopy can be improved using the SERS method, which can be applied on either a colloidal sample or solid support [23], with the former technique being more applicable for the development of an assay or even for in vivo monitoring in the blood. Thus, the aim of this work was to develop and apply the SERS technique to study the encapsulation of drugs by sugammadex (**SDX**) in an aqueous medium. First, the properties of the silver colloid were tested and accordingly modified in order to observe the efficient enhancement of the Raman scattering of sugammadex and the drugs. Then, concentration-dependent SERS spectra of sugammadex and drugs were obtained, assigned, and associated with their molecular structures. To observe the SERS spectra of the sugammadex/drug complexes, different preparation procedures of the measuring sample were used. The observed spectral differences indicated the mechanism of drug encapsulation within the sugammadex cavity as well as types of host–guest interactions. The most important goal is to collect all data in biorelevant concentrations of components, not exceeding the micromolar concentration range.

## 2. Results and Discussion

### 2.1. Raman Spectrum of Sugammadex

**Figure 2:**
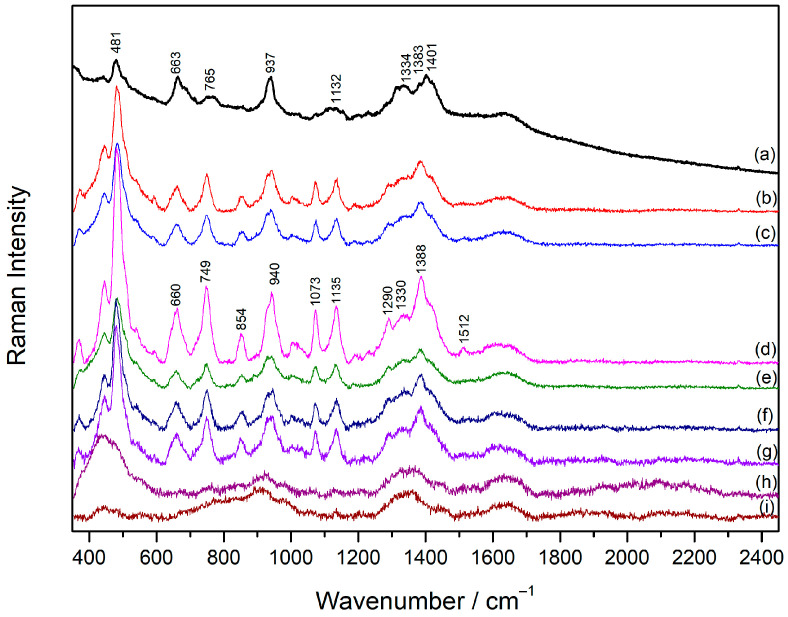
(**a**) The Raman spectrum of **SDX** solution (1 × 10^−2^ M) and the SERS spectra of **SDX**: (**b**) 1 × 10^−4^ M, (**c**) 5 × 10^−5^ M, (**d**) 1 × 10^−5^ M, (**e**) 5 × 10^−6^ M, (**f**) 1 × 10^−6^ M, (**g**) 5 × 10^−7^ M, (**h**) 1 × 10^−7^ M, and (**i**) the silver colloid. The spectra are displaced for visual clarity.

There are no studies specifically focused on the Raman spectrum of sugammadex; however, general characterization is necessary for further studies by the SERS technique. The Raman spectrum of **SDX** solution (5 × 10^−2^ M) was dominated by bands associated with vibrational modes of glucose: deformations of CH groups (1334 and 1383 cm^−1^), symmetrical stretching of C–O–C bonds (937 cm^−1^), in-plane deformation (765 cm^−1^), and out-of-plane deformation (481 cm^−1^) of the glucose ring (Figure 2a, Table 1) [24,25]. Bands at 1115 and 1132 cm^−1^ were assigned to the stretching vibrations of the C–C bond, while the intense band at 663 cm^−1^ was attributed to the stretching of the C–S bond [26].

### 2.2. SERS Spectra of Sugammadex

#### 2.2.1. Aggregation of the Silver Colloid with Inorganic Salts

**Figure 3:**
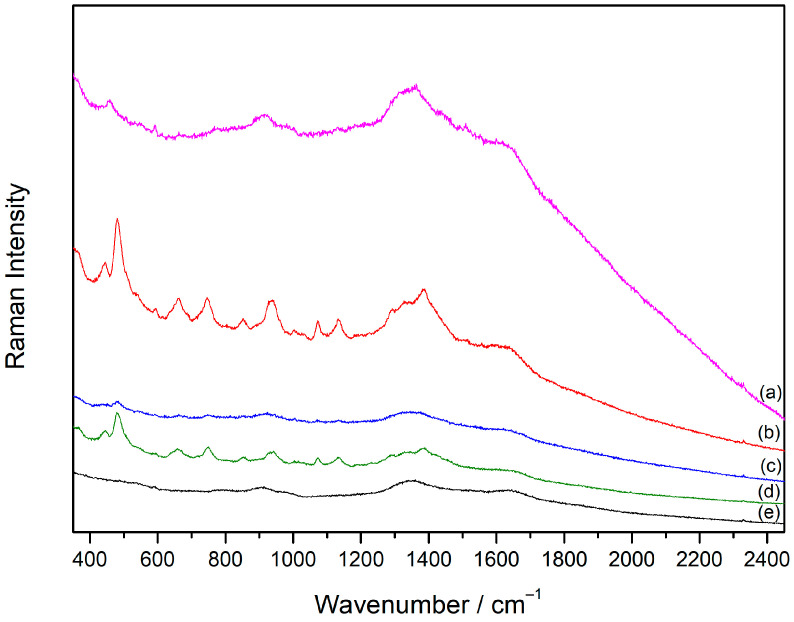
SERS spectra of **SDX** (1 × 10^−5^ M) in the silver colloid aggregated with inorganic salts: (**a**) KCl, (**b**) Ca(NO_3_)_2_, (**c**) CaCl_2_, (**d**) NaNO_3_, and (**e**) NaCl; *c*(salt) = 5 × 10^−4^ M. The spectra are displaced for visual clarity.

The prerequisite for the enhancement of the Raman scattering is an efficient metal substrate [27]. Silver nanoparticles stabilized by chloride ions on their surface (AgNP) were used as the SERS substrate in this research. By mixing the prepared silver colloid with the cyclodextrin solution, vibrational bands of **SDX** were not observed, and the obtained spectrum resembled the Raman spectrum of the silver colloid alone (Figure S1). It was assumed that the **SDX** molecules were not adsorbed on the metal nanoparticles due to repulsive electrostatic interactions between the anionic layer of the chloride ions on the silver surface and deprotonated carboxylate groups in the **SDX** molecules. To stimulate the adsorption of **SDX** onto the silver nanoparticles, properties of the metal surface were modified by the addition of inorganic salts. Inorganic salts are often used as aggregating agents in SERS spectroscopy since they promote the aggregation of metal nanoparticles and the formation of SERS-active sites (“hot spots”) [28]. At the same time, they partially neutralize the charge of the ions on the nanoparticle surface, facilitating the adsorption of the molecules of the same charge. In order to find the efficient aggregating agent for the SERS study of cyclodextrin, the spectra of **SDX** solution (1 × 10^−5^ M) were measured in the silver colloid after the addition of the following inorganic salts: NaCl, KCl, CaCl_2_, Ca(NO_3_)_2_, and NaNO_3_ (Figure 3). The final concentration of inorganic salts in the measuring samples was 5 × 10^−4^ M. When chloride-containing salts were added into the colloidal sample with **SDX**, the characteristic bands of **SDX** molecules were not observed. However, when nitrate salts were used as aggregating agents, the SERS spectrum of **SDX** was obtained. Given that chloride ions have a higher affinity for silver than nitrate ions [29], it was assumed that they interacted with the metal surface or were located close to the silver nanoparticles, thus hindering the access of the **SDX** molecules to the nanoparticle surface. When silver nanoparticles were aggregated using the nitrate salts, the SERS spectrum of **SDX** in the colloidal sample aggregated with Ca(NO_3_)_2_ was more intense than in the sample aggregated with NaNO_3_. This was consistent with the findings that salts containing multiple charged cations are more effective aggregating agents for the adsorption of negatively charged molecules on the metal nanoparticles than the salts containing singly charged cations [30].

**Table 1:** Assignment of the relevant vibrational bands in the Raman and SERS spectra of sugammadex [24,25,26,31].

Wavenumber/cm^−1^		Assignment
Raman	SERS	
	1512	*ν*_asym_ COO^–^
1401		*δ* CH
1383	1388	*δ* CH
1334	1330	*δ* CH, *δ* CH_2_
1311	1316	*δ* CH, *δ* CH_2_
	1290	*ν*_sym_ COO^–^
1132	1135	*ν* C–C
1115		*ν* C–C
1074	1073	*ν* C–O
	1014	*ν*_sym_ C–O–C
937	940	*ν*_sym_ C–O–C
	854	*δ* ring “breathing” glucose
765	749	*δ*_ip_ ring glucose
663	660	*ν* C–S
481	484	*δ*_oop_ ring glucose
440	444	*δ*_oop_ OH

Abbreviations: *ν*, stretching; *δ*, deformation; sym, symmetric; asym, asymmetric; ip, in-plane; oop, out-of-plane.

#### 2.2.2. Concentration-Dependent SERS Spectra of Sugammadex

Given that Ca(NO_3_)_2_ proved to be an effective aggregating salt for AgNP, concentration-dependent SERS spectra of **SDX** were recorded in the aggregated silver colloid in the concentration range 1 × 10^−7^–1 × 10^−4^ M (Figure 2b–h). If compared to the Raman spectrum of **SDX**, more intense and defined vibrational bands were observed in the **SDX** SERS spectrum (1 × 10^−4^ M), implying an ordered arrangement of the **SDX** molecules on the silver nanoparticle surface. The selective enhancement of the bands originating from bond stretching and glucose ring deformations (1135, 1073, 1014, 854, 749, 484 cm^−1^) was obtained (Table 1). In addition, new bands, observed at 1290 and 1512 cm^−1^ in the SERS spectrum, were attributed to the symmetric and antisymmetric stretching vibration of the carboxylate group [31], respectively, indicating the orientation of the **SDX** molecules towards the silver nanoparticles with carboxylate groups close to the metal surface. This supported the fact that the calcium ions neutralized the negative charge of the silver nanoparticles, and at the same time they electrostatically attracted negatively charged **SDX** molecules close to the metallic surface. A decrease in the **SDX** concentration resulted in non-linear changes in the overall SERS spectra intensity, with the SERS spectrum at the **SDX** concentration of 1 × 10^−5^ M being the most intense. It was assumed that at this concentration, complete coverage of the metal surface was achieved with the molecules optimally oriented towards the nanoparticle surface, resulting in the strongest enhancement of the Raman scattering. The **SDX** molecules were detected by SERS at a concentration as low as 5 × 10^−7^ M.

### 2.3. Raman Spectra of Vecuronium and Rocuronium

The Raman spectra of the solid substances and aqueous solutions (1 × 10^−2^ M) of the drugs, Vecuronium bromide and Rocuronium bromide, were measured.

**Figure 4:**
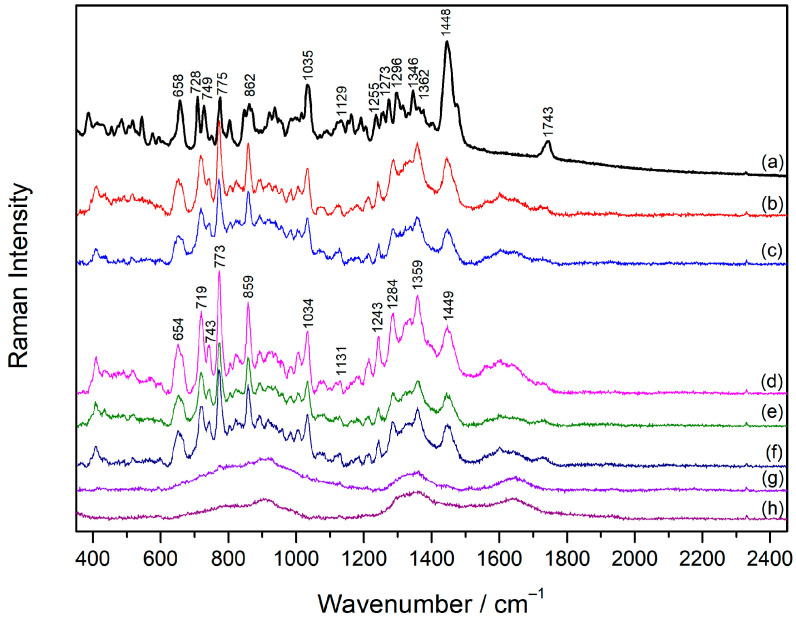
(**a**) The Raman spectrum of solid **Vec** and the SERS spectra of **Vec**: (**b**) 1 × 10^−4^ M, (**c**) 5 × 10^−5^ M, (**d**) 1 × 10^−5^ M, (**e**) 5 × 10^−6^ M, (**f**) 1 × 10^−6^ M, (**g**) 5 × 10^−7^ M, and (**h**) the silver colloid. The spectra are displaced for visual clarity.

The Raman spectrum of solid **Vec** was rich in bands, especially in the spectral region below 1500 cm^−1^ (Figure 4a, Table 2). The most intense band in the spectrum at 1448 cm^−1^ originated from the deformations of CH_2_ and CH_3_ groups, characteristic of saturated steroids [32,33]. Most of the bands in the spectral range between 1150 and 1450 cm^−1^ were attributed to the CH_2_ and CH_3_ deformation modes of the piperidine and androstane moieties in the **Vec** molecular structure [32,34]. The band at 1035 cm^−1^ corresponded to the skeletal stretching vibration of piperidine [34], while piperidine CH_2_ deformation mode gave rise to the band at 1163 cm^−1^ [35]. The “breathing” vibrations of the cyclopentane and cyclohexane steroid rings were obtained at 728, 775, and 862 cm^−1^. The ester groups in the molecule contributed to the bands at 1743 and 1129 cm^−1^, which were assigned to C=O stretching and C–O stretching, respectively.

**Figure 5:**
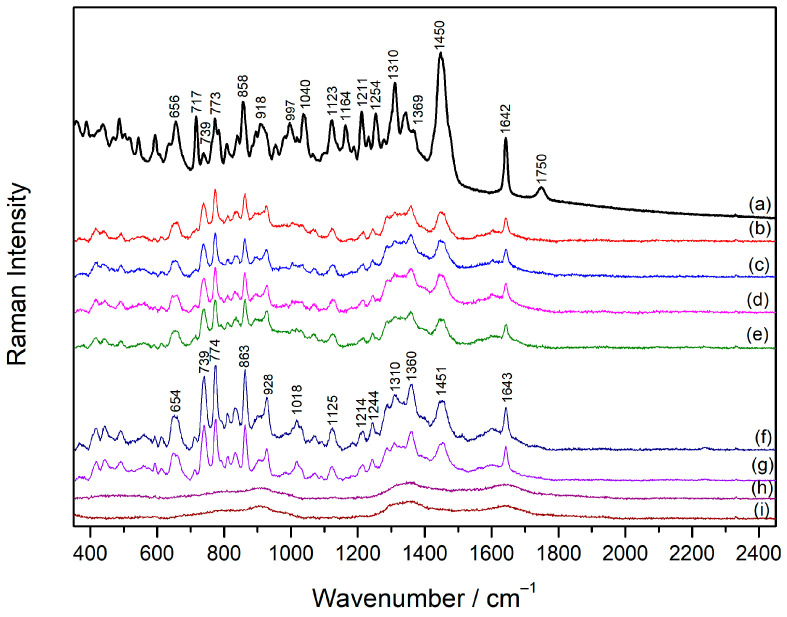
(**a**) The Raman spectrum of solid **Roc** and the SERS spectra of **Roc**: (**b**) 1 × 10^−4^ M, (**c**) 5 × 10^−5^ M, (**d**) 1 × 10^−5^ M, (**e**) 5 × 10^−6^ M, (**f**) 1 × 10^−6^ M, (**g**) 5 × 10^−7^ M, and (**h**) 5 × 10^−7^ M and (**i**) the silver colloid. The spectra are displaced for visual clarity.

Similarly to **Vec**, the most intense band in the Raman spectrum of solid **Roc**, characteristic of steroids, was obtained at 1450 cm^−1^ (Figure 5a, Table 2). The bands in the spectral range between 1170 and 1380 cm^−1^ were attributed to CH_2_ deformation vibrations of androstane, pyrrole, and morpholine units in the molecular structure of the studied drug. The band typical for morpholine ring stretching was noted at 1040 cm^−1^ [36,37], while pyrrole and morpholine vibrations contributed to the band at 839 cm^−1^ [38,39]. Pyrrole CH deformations gave rise to the band at 1123 cm^−1^. The weak band at 1750 cm^−1^ originated from the C=O stretching vibration of the ester group, while the band at 1642 cm^−1^ was the result of the C=C stretching of the substituent on the pyrrole ring.

Using the excitation wavelength at 785 nm, the Raman spectra of the aqueous solution of the drugs (1 × 10^−2^ M) were not obtained (Figure S2). The lack of Raman spectra was attributed to the low sensitivity of the Raman spectroscopy and poorly polarizable structures of the studied molecules.

### 2.4. Concentration-Dependent SERS Spectra of Vecuronium and Rocuronium

The SERS spectra of **Vec** were measured in the concentration range 5 × 10^−7^–1 × 10^−4^ M in the silver colloid not containing the aggregating agent (Figure 4, Table 2). The spectra obtained in the concentration range 1 × 10^−6^–1 × 10^−4^ M resembled each other concerning the band position and intensity, indicating that the change in concentration did not induce changes in the orientation of the molecules towards the metal surface. Slightly more defined bands were observed in the **Vec** spectrum at a concentration of 1 × 10^−5^ M, while the concentration of 1 × 10^−6^ M was the lowest one at which **Vec** was detected. Most of the SERS bands originated from androstane vibrational modes [32,33], among which were the intense ones at 773, 859, 1359, and 1449 cm^−1^. The stretching vibration of the piperidine ring contributed to the band at 1034 cm^−1^, while CH_2_ deformation gave rise to the band noted at 1179 cm^−1^ [35]. The observed piperidine and steroid bands in the **Vec** SERS spectra pointed to the adsorption mechanism of the drug molecules on the silver surface. Hence, the adsorption was most likely driven by electrostatic attractive interactions between the negatively charged chloride ions on the silver surface and the positively charged piperidine moiety. This was supported by a significant change in the zeta potential of the silver nanoparticles upon the addition of the drugs into the colloidal suspension. If compared to the negative zeta potential of the silver nanoparticles of −35.97 mV, the positive values of the zeta potential of 11.34 mV and 10.65 mV were measured for the silver colloid containing Vecuronium and Rocuronium, respectively (Figures S3 and S4). The change in the surface charge of the nanoparticles indicated electrostatic binding between the negative ions on the silver surface and the positively charged parts of the drug molecules.

The SERS spectra of **Roc** were measured in the concentration range of 1 × 10^−7^–1 × 10^−4^ M (Figure 5, Table 2). Although the most intense SERS spectrum was observed at a concentration of 1 × 10^−6^ M, variations in the drug concentration did not result in notable spectral changes, implying similar placement of the drug molecules on the metal surface regardless of the **Roc** concentration. Similarly to the **Vec** SERS spectra, the most intense bands in the **Roc** SERS spectrum (1 × 10^−6^ M) originated from androstane vibrational modes (774, 863, 1360, and 1451 cm^−1^) [32,33]. The bands attributed to the pyrrole ring deformation at 1214 cm^−1^ and CH deformation modes at 1125 cm^−1^ pointed to the pyrrole ring facing the silver surface [38]. This was supported by the appearance of the C=C stretching band at 1643 cm^−1^, associated with the substituent on the pyrrole ring, and a lack of the band at 1040 cm^−1^, characteristic of morpholine. It was assumed that the positively charged pyrrole ring was electrostatically attracted to the negatively charged silver surface when the **Roc** molecules were oriented with the pyrrole ring close to and the morpholine moiety distant from the silver nanoparticles. The detection limit for **Roc** under the applied experimental conditions was 5 × 10^−7^ M.

### 2.5. SERS Spectra of Sugammadex/Drug Complexes

To better understand the interactions between cyclodextrin and the drugs, **SDX**/drug complexes were prepared at a 1:1 molar ratio using a silver colloidal suspension. The drug concentration producing the most intense SERS signal was selected for complex preparation. Specifically, the concentrations for the **SDX**/**Vec** complex were set at 1 × 10^−5^ M for both components, while for the **SDX**/**Roc** complex, concentrations of 1 × 10^−6^ M were used. Initially, the SERS spectra of the **SDX**/**Vec** mixtures showed no changes immediately after mixing, displaying only a combination of the individual spectra (Figure S5). Consequently, a 30 min room temperature incubation was incorporated into the preparation process.

To assess whether the sequence of the component addition influenced Raman scattering enhancement, complexes were prepared using three different methods: (A) the drug was added to the silver colloid and incubated for 30 min before introducing **SDX**; (B) **SDX** was added to the silver colloid and incubated for 30 min before the drug was added; and (C) **SDX** and the drug were mixed and incubated for 30 min prior to adding the silver colloid. Spectra were recorded immediately after preparation and again following a 30 min incubation.

**Figure 6:**
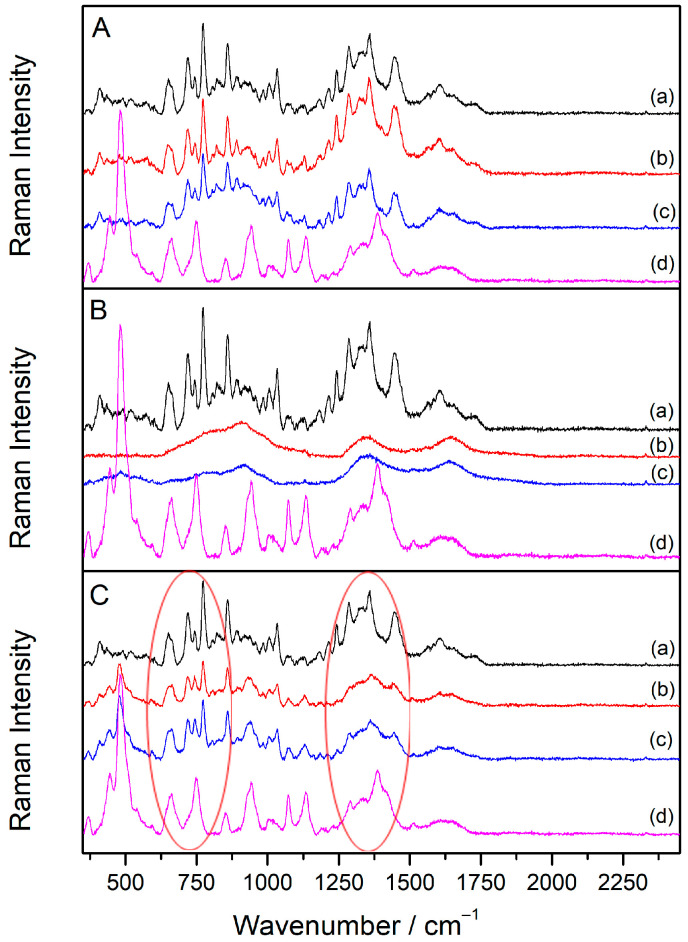
The SERS spectra of **SDX**/**Vec** mixtures (*c*(**SDX**) = *c*(**Vec**) = 1 × 10^−5^ M) prepared by methods **A**, **B** and **C**. The SERS spectra: (**a**) *c*(**Vec**) = 1 × 10^−5^ M, (**b**) **SDX**/**Vec** mixture measured immediately after preparation, (**c**) **SDX**/**Vec** mixture measured 30 min after preparation, (**d**) *c*(**SDX**) = 1 × 10^−5^ M. The spectra are displaced for visual clarity. Red ovals denote areas relevant for the discussion of the results.

**Figure 7:**
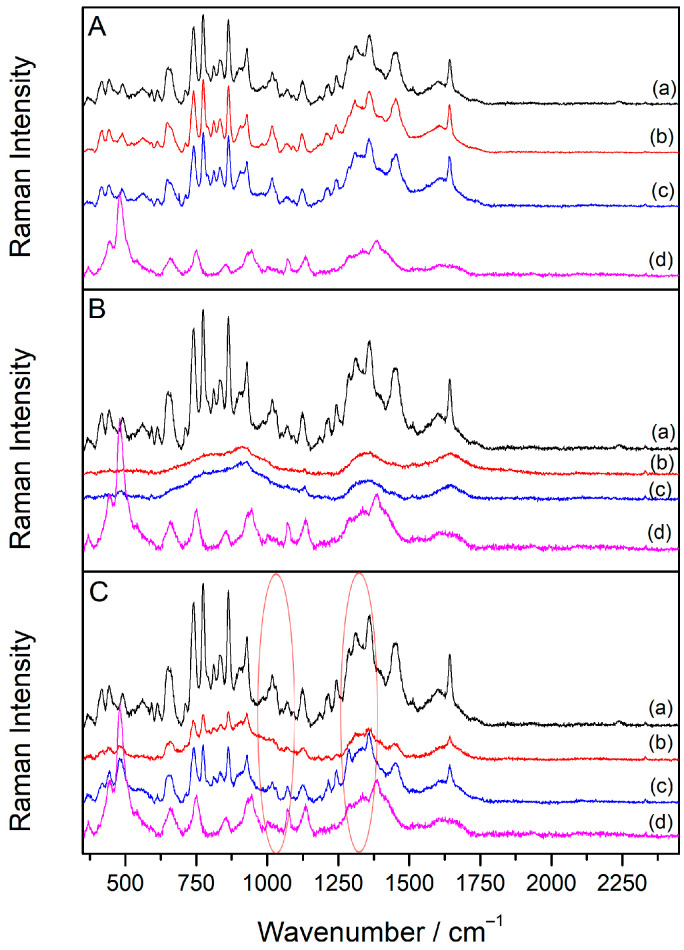
The SERS spectra of **SDX**/**Roc** mixtures (*c*(**SDX**) = *c*(**Roc**) = 1 × 10^−6^ M) prepared by methods **A**, **B** and **C**. The SERS spectra: (**a**) *c*(**Roc**) = 1 × 10^−6^ M, (**b**) **SDX**/**Roc** mixture measured immediately after preparation, (**c**) **SDX**/**Roc** mixture measured 30 min after preparation, (**d**) *c*(**SDX**) = 1 × 10^−6^ M. The spectra are displaced for visual clarity. Red ovals denote areas relevant for the discussion of the results.

For the **SDX**/**Vec** mixture prepared by method A, the SERS spectra recorded immediately and after 30 min closely resembled the spectrum of **Vec** alone (Figure 6A), with no characteristic **SDX** bands observed. This indicates that positively charged **Vec** molecules strongly adsorbed onto the silver nanoparticles likely due to electrostatic attraction to the chloride anions on the nanoparticle surface. As a result, **Vec** did not bind to **SDX**, and **SDX** was unable to approach the nanoparticle surface. A similar observation was made for the **SDX**/**Roc** mixture prepared using method A (Figure 7A).

In the SERS spectra of the **SDX**/**Vec** mixture prepared using method B—where cyclodextrin was incubated with the silver colloid for 30 min before adding the drug—no bands corresponding to **SDX** or **Vec** were detected, either immediately after the preparation or after 30 min (Figure 6B). This outcome is likely due to the negatively charged **SDX** molecules being repelled by the anionic surface layer of the nanoparticles, preventing their adsorption onto the silver surface. Instead, **SDX** remained in the solution, where it formed a complex with the added drug but stayed too far from the metal surface to enable Raman signal enhancement. A similar result was observed for the **SDX**/**Roc** mixture prepared using method B (Figure 7B).

**Figure 8:**
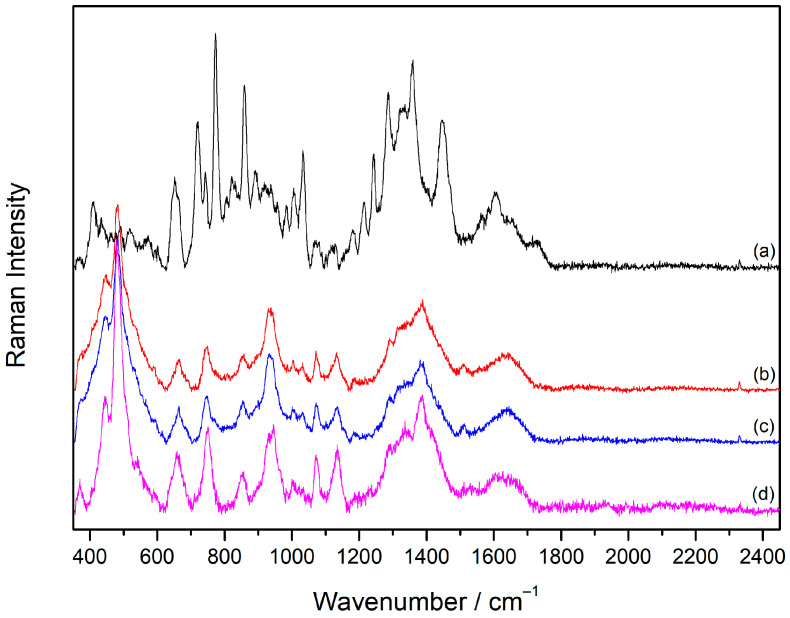
SERS spectra: (**a**) *c*(**Vec**) = 1 × 10^−5^ M, (**b**) **SDX**/**Vec** mixture prepared by method B in the silver colloid aggregated with Ca(NO_3_)_2_ measured immediately after addition of **Vec**, (**c**) **SDX**/**Vec** mixture prepared by method II in the silver colloid aggregated with Ca(NO_3_)_2_ measured 30 min after addition of **Vec**, and (**d**) *c*(**SDX**) = 1 × 10^−5^ M. The spectra are displaced for visual clarity.

To enhance **SDX** adsorption onto silver nanoparticles, method B was modified by incubating **SDX** with the silver colloid in the presence of the aggregating salt Ca(NO_3_)_2_ (5 × 10^−4^ M) for 30 min before adding **Vec** (Figure 8). Interestingly, the resulting spectra showed bands corresponding to **SDX** but none for **Vec**. The addition of the inorganic salt caused silver nanoparticle aggregation, creating SERS-active sites and facilitating **SDX** adsorption onto the metal surface. However, there was no spectral evidence of interactions between the adsorbed **SDX** molecules and the subsequently added **Vec**, indicating that the cyclodextrin/drug complex did not form on the silver surface.

In contrast, the SERS spectra of **SDX**/drug mixtures prepared using method C—where the **SDX**/drug complex was pre-formed and incubated for 30 min before introducing the silver colloid—exhibited notable spectral differences compared to the individual spectra of **SDX** or the drugs alone (Figure 6C, Table 2). For the **SDX**/**Vec** mixture, the intensity in the 1150–1400 cm^−1^ region, associated with methyl and methylene deformation bands, decreased. Additionally, steroid bands of **Vec** shifted from 1449 to 1445 cm^−1^ and from 1359 to 1365 cm^−1^, while the glucose band of **SDX** shifted from 484 to 481 cm^−1^. These spectral changes, primarily involving the glucose rings of **SDX** and the steroid rings of **Vec**, suggest hydrophobic interactions between **Vec** and **SDX**, consistent with the drug being encapsulated within the cyclodextrin cavity. This method of binding molecules to cyclodextrins is well documented in the literature [40,41].

The SERS spectra of the **SDX**/**Roc** mixture prepared using method C closely resembled the spectrum of the drug alone (Figure 7C, Table 2). Only the **SDX**-specific bands at 484 and 444 cm^−1^ were detected, while all other bands originated from **Roc** vibrational modes, further supporting the strong affinity of **Roc** molecules for the metal substrate. However, the absence of the morpholine band at 1040 cm^−1^ in the mixture spectrum, along with the shift of the steroid band from 1451 to 1455 cm^−1^, suggested a binding mechanism between **Roc** and **SDX**. This interaction was likely driven by electrostatic attraction between the positively charged pyrrole group of **Roc** and the negatively charged carboxylate groups of **SDX**. The charged portions of the complex were positioned near the SERS substrate, while hydrophobic interactions occurred between the androstane core and **SDX** glucose units. Meanwhile, the morpholine ring was oriented outside the cavity, away from the metal surface.

**Figure 9:**
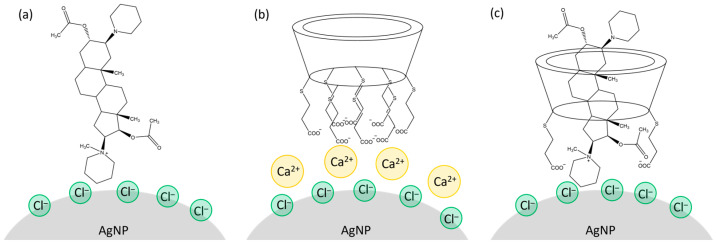
Scheme of adsorption of (**a**) **Vec**, (**b**) **SDX**, and (**c**) **SDX**/**Vec** complex onto the silver nanoparticle surface.

The SERS spectra obtained from samples prepared using different component addition sequences revealed that **SDX**’s interactions with the silver nanoparticle surface were stronger than its interactions with the drugs. For both **Roc** and **Vec**, the SERS spectra of the **SDX**/drug complex appeared only when the complex was pre-formed before adding the silver colloid. The similarity between these complex spectra and the spectra of the individual drugs suggests that the drugs likely oriented their charged piperidine and pyrrole units toward the silver surface. A similar orientation is inferred for the drugs within the **SDX** cavity (Figure 9). Furthermore, the resemblance of the SERS spectra of the **SDX**/drug complex to that of **SDX** in the aggregated colloid indicates that **SDX** likely positions its carboxylate groups toward the silver nanoparticle surface when hosting a drug molecule in its cavity.

## 3. Materials and Methods

### 3.1. Chemicals and Solutions

Silver nitrate (Gram-Mol d.o.o, Zagreb, Croatia), sodium hydroxide (Kemika d.d., Zagreb, Croatia), hydroxylamine hydrochloride (Kemika d.d., Zagreb, Croatia), sodium chloride (Kemika d.d., Zagreb, Croatia), potassium chloride (Kemika d.d., Zagreb, Croatia), calcium chloride (Kemika d.d., Zagreb, Croatia), calcium nitrate (Kemika d.d., Zagreb, Croatia), and potassium nitrate (Kemika d.d., Zagreb, Croatia) were of p.a. purity and used as received. Vecuronium bromide and Rocuronium bromide were the highest purity as standards obtained from US. Pharmacopeia, 12,601 Twinbrook Parkway, Rockville, MD, USA, while sugammadex (Bridion^®^, Merck Sharp & Dohme Limited, Hertfordshire, UK) was kindly provided by Medika d.d., Zagreb, Croatia.

Water was purified by passage through Milli-Q (Millipore, Merck KGaA, Darmstadt, Germany) deionization and filtration columns. Stock solutions of Vecuronium bromide and Rocuronium bromide (1 × 10^−2^ M) were prepared by dissolving the solid substance in Milli-Q water.

### 3.2. Colloids Preparations

Silver colloidal nanoparticles (AgNP) were synthesized according to a previously described method [42]. Firstly, 17 mg of solid silver nitrate was dissolved in 90 mL of Milli-Q water. The hydroxylamine hydrochloride solution was prepared by dissolving 17 mg of hydroxylamine hydrochloride in 10 mL of Milli-Q water, and in the solution 150 μL of sodium hydroxide solution (2 M) was added. The prepared alkaline solution of hydroxylamine hydrochloride was mixed with silver nitrate solution under vigorous stirring, and the resulting suspension was left to stir for another 10 min. The prepared colloidal suspension was grayish yellow, characterized by an absorption maximum at 406 nm and a pH value of 6.67. The measured value of the zeta potential was −35.97 mV. To reveal the size and shape of the prepared silver nanoparticles, TEM images were taken (Figure S6), showing the uniformly shaped silver nanospheres with a diameter of 20–120 nm.

All glassware used for synthesis was thoroughly cleaned with a detergent solution, rinsed with 5% (*v*/*v*) nitric acid, and at the end with water of Milli-Q purity.

### 3.3. Samples’ Preparation

The sample of **SDX** (1 × 10^−5^ M) for the SERS measurement was prepared by mixing the **SDX** solution (15 μL) with the silver colloid (105 μL). In order to test inorganic salts as aggregating agents, the samples of **SDX** were prepared by the addition of an inorganic salt (10 μL) into the mixture of the sliver colloid (105 μL) and **SDX** (5 μL). The following inorganic salts were used: NaCl, KCl, CaCl_2_, Ca(NO_3_)_2_, and NaNO_3_, and the final concentration of the salt in the sample was 5 × 10^−4^ M.

For the concentration-dependent SERS measurements of **SDX**, samples were prepared in the aggregated silver colloid by mixing the silver colloid (105 μL) and **SDX** stock solution (5 μL), followed by the addition of Ca(NO_3_)_2_ (10 μL). The final concentrations of **SDX** in the measuring samples were 1 × 10^−7^, 5 × 10^−7^, 1 × 10^−6^, 5 × 10^−6^, 1 × 10^−5^, 5 × 10^−5^, and 1 × 10^−4^ M, and the concentration of Ca(NO_3_)_2_ was 5 × 10^−4^ M. The samples were incubated at room temperature for 15 min prior to the SERS measurements.

The samples of **Vec** and **Roc** for the concentration-dependent SERS measurements were prepared by mixing the silver colloid (105 μL) and stock solutions of the drugs (15 μL). The final concentrations of the drugs in the SERS samples were 1 × 10^−7^, 5 × 10^−7^, 1 × 10^−6^, 5 × 10^−6^, 1 × 10^−5^, 5 × 10^−5^, and 1 × 10^−4^ M. The SERS spectra were acquired immediately after the sample preparation.

The samples of **SDX**/drug mixtures were prepared in the **SDX**/drug molar ratio of 1/1 by mixing the silver colloid (105 μL), the drug (10 μL), and **SDX** (5 μL). In the **SDX**/**Vec** and **SDX**/**Roc** measuring samples, the final concentration of the components was 1 × 10^−5^ M and 1 × 10^−6^ M, respectively. Each mixture was prepared in three different ways according to the order of the addition of the components: (A) the drug was added to the AgNP colloid and the mixture was kept for 30 min at room temperature, followed by the addition of **SDX** to the mixture; (B) **SDX** was added to the AgNP colloid and the mixture was kept for 30 min at room temperature, followed by the addition of the drug to the mixture; (C) and the **SDX**/drug complex was prepared and kept for 30 min at room temperature, followed by the addition of the AgNP colloid to the mixture. The spectra of the samples were measured immediately after preparation and 30 min later.

In addition, a sample of the **SDX**/**Vec** mixture was prepared in the aggregated silver colloid by method (B). In this case, Ca(NO_3_)_2_ was mixed with the silver colloidal suspension containing **SDX** before the addition of **Vec**.

### 3.4. Instrumentation

Raman and SERS spectra were measured using a inVia Raman microscope (Renishaw plc, Gloucestershire, UK) with laser excitation at 785 nm. The ×5 microscopic objective (NA = 0.12) was used for the measurements. The spectra were acquired for solid samples in aluminum holders and for liquid samples in aluminum pans (V = 40 μL, OD 5.4 mm × H 2.6 mm). Measurement conditions, such as applied laser power and exposure time, depended on the measuring sample. Therefore, the Raman spectra of stock solutions were recorded at a laser power of 319 mW, while the solid substances were measured using a laser power of 159 mW. The samples were illuminated with laser radiation for 10 s during the Raman measurements. The concentration-dependent SERS spectra of sugammadex, the drugs, and the sugammadex/drug complexes were acquired at 32 mW laser power and 60 s exposure time. All the SERS spectra were baseline-corrected using Wire software 5.3.

The plasmon absorption wavelength of the silver colloidal suspension was measured using the spectrometer (model SPECORD 200, Analytik Jena GmbH+Co. KG, Jena, Germany). Quartz cells of 2 mm path length were used for the measurements.

pH measurements were performed on a pH meter (model MP 220, Mettler Toledo GmbH, Giessen, Germany) with a Mettler Toledo InLab 413 combined glass calomel electrode. The pH meter was calibrated using standard aqueous buffer solutions of pH 7.00 and 4.01.

Zeta potential measurements were taken using a Zetasizer Nano ZS (Malvern Panalytical Ltd., Malvern, UK) set-up.

Transmission electron micrographs (TEMs) were taken on a Zeiss EM10 transmission electron microscope (Carl Zeiss, Leipzig, Germany) from a drop of a colloid on a carbon-coated grid (copper, 100 mesh).

## 4. Conclusions

Surface-enhanced Raman scattering spectroscopy was successfully used to obtain a more complete insight into the way sugammadex interacted with the studied drug molecules. Negatively charged silver colloidal nanoparticles proved to be an adequate SERS substrate for the structural characterization of positively charged Vecuronium and Rocuronium in the biorelevant micromolar concentration range. However, the SERS enhancement of negatively charged sugammadex (**SDX**) was observed only after silver nanoparticle aggregation, with calcium nitrate proving to be the most effective aggregating agent.

For a more detailed study of interactions between sugammadex (**SDX**) and the drugs, **SDX**/drug complexes were prepared by mixing Vecuronium or Rocuronium with sugammadex in a 1/1 molar ratio at biorelevant (micromolar) concentrations of the components. Given the high binding constants between **SDX** and these drugs, full complexation was assumed [10]. The complexes were prepared using three methods, varying the order of component addition and incubation time. SERS spectra of the **SDX**/drug complexes were obtained only when the **SDX** and drug were incubated together for 30 min prior to adding the silver colloid. The observed SERS bands of the charged moieties implied that the insertion of the drug molecule into the **SDX** cavity was driven by electrostatic interactions between the positively charged nitrogen-containing drug rings and the negatively charged carboxylate **SDX** groups. Additionally, spectral changes in the **SDX** glucose rings and drug steroid rings confirmed that the encapsulated drug was stabilized within the **SDX** cavity through hydrophobic interactions between the host **SDX** and the guest drug molecule.

## Figures and Tables

**Table 2:** Assignment of the relevant vibrational bands in the Raman and SERS spectra of Vecuronium and Rocuronium and their complexes with sugammadex [26,31,32,33,34,35,36,37,38,39].

Wavenumber/cm^−1^						Assignment
Raman	SERS		Raman	SERS		
Vec	Vec 10^−5^ M	SDX/Vec	Roc	Roc 10^−6^ M	SDX/Roc	
1743			1750			ν C=O ester
			1642	1643	1643	ν C=C chain
			1450	1451	1455	δ_sc_ CH_2_, δ_as_ CH_3_ (steroid, morpholine)
1448	1449	1445				δ_sc_ CH_2_, δ_as_ CH_3_ (steroid, piperidine)
1362	1359	1365	1369	1360	1359	δ_wg_ CH_2_, δ_s_ CH_3_
1346						δ_wg_ CH_2_, δ_s_ CH_3_
			1310	1310		δ_wg_ CH_2_ (steroid, morpholine)
1296	1284	1285				δ_wg_ CH_2_ (steroid, piperidine)
1273			1278	1285	1285	δ_wg_ CH_2_ (steroid)
1255	1243	1243	1254	1244	1243	δ_tw_ CH_2_
1234	1214	1212	1232			δ_tw_ CH_2_
			1211	1214	1215	δ ring pyrrole
1206						δ_tw_ CH_2_
1192			1189			δ_rc_ CH_2_
			1164			δ_rc_ CH_2_ (morpholine)
1163	1179	1185				δ_rc_ CH_2_ (piperidine)
1129	1131	1131				ν C–O (**Vec**), ν C–C (**SDX**)
			1123	1125	1125	δ CH pyrrole
			1066 w	1070	1072	ν C–C, δ CH (pyrrole)
			1040			ν C–C (morpholine)
1035	1034	1034				ν C–C (piperidine)
				1018		δ ring pyrrole
			997			ν C–C (morpholine)
		937				*ν*_sym_ C–O–C (**SDX**)
			918 w	928	928	δ ring pyrrole
862	859	859	858	863	863	cyclopenthane ring “breathing”
			839	835	832	ν C–C, ν C–O (morpholine), δ ring pyrrole
775	773	774	773	774	774	cyclohexane ring “breathing”
749	743	743	739	739	740	cyclohexane ring “breathing”
728	719	719	717	713 w	714 w	cyclohexane ring “breathing”
658	654	656	656	654	661	δ skeleton steroid
		481			484	δ_oop_ ring glucose (**SDX**)
		441			444	δ_oop_ OH (**SDX**)

Abbreviations: ν, stretching; δ, deformation; s, symmetric; as, asymmetric; ip, in-plane; oop, out-of-plane; sc, scissoring; wg, wagging; tw, twisting; rc, rocking; w, weak.

## Data Availability

All data are included in the article.

## Supplementary Materials

The following supporting information can be downloaded at: https://www.mdpi.com/article/10.3390/molecules30020231/s1, Figure S1: The SERS spectrum of **SDX** (1 × 10^−5^ M) in the silver colloid not containing an aggregating agent; Figure S2: Raman spectra of the drug stock solutions (1 × 10^−2^ M); Figure S3: Zeta potential distribution for colloid alone (black line) and colloid with added Vecuronium (red line); Figure S4: Zeta potential distribution for colloid alone (black line) and colloid with added Rocuronium (red line); Figure S5: The SERS spectra of **SDX**/**Vec** mixtures (*c*(**SDX**) = *c*(**Vec**) = 1 × 10^−5^ M) differing in the order of the addition of components according to methods A, B, and C and recorded immediately after the last component was added. The SERS spectra: (a) *c*(**Vec**) = 1 × 10^−5^ M, (b) **SDX**/**Vec** mixture prepared by method A, (c) **SDX**/**Vec** mixture prepared by method B, (d) **SDX**/**Vec** mixture prepared by method C, and (e) *c*(**SDX**) = 1 × 10^−5^ mol/L. The spectra are displaced for visual clarity; Figure S6: TEM micrograph of colloidal nanoparticles.
