# Supplementary material for: Encapsulation of Vecuronium and Rocuronium by Sugammadex Investigated by Surface-Enhanced Raman Spectroscopy
Source: Molecules. 2025 Jan 9;30(2):231. doi: 10.3390/molecules30020231 (PMC11767325; doi:10.3390/molecules30020231)
Supplement: Supplementary file 1 [file molecules-30-00231-s001.zip › molecules-3343351-supplementary.pdf]

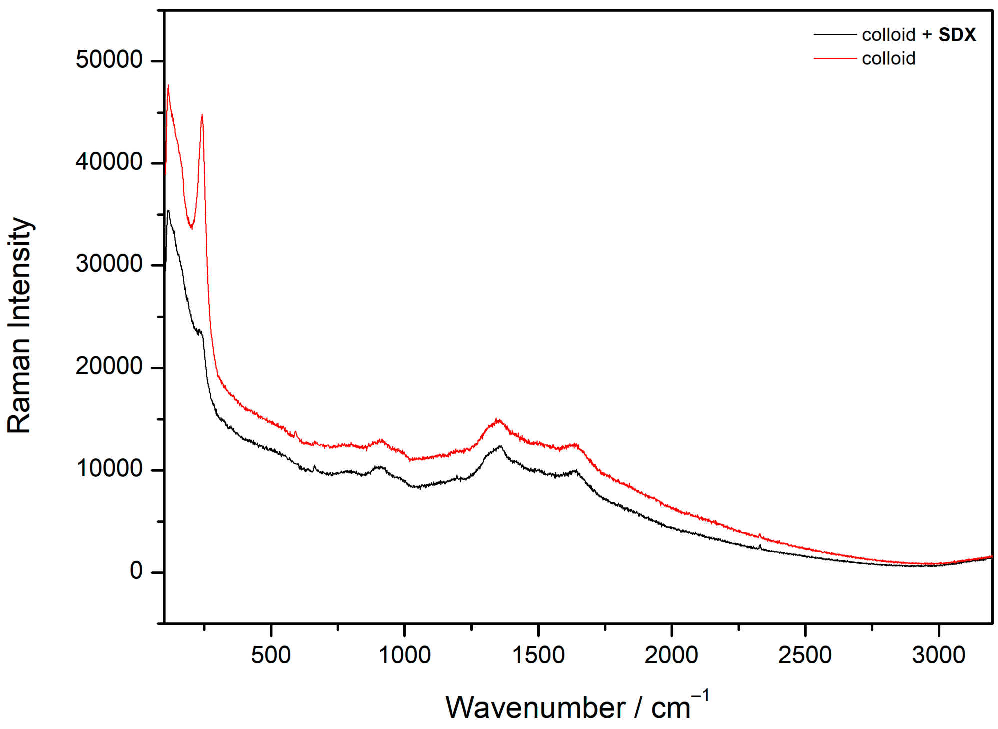

Figure S1. The SERS spectrum of **SDX** (1×10^{-5} M) in the silver colloid not containing an aggregating agent.

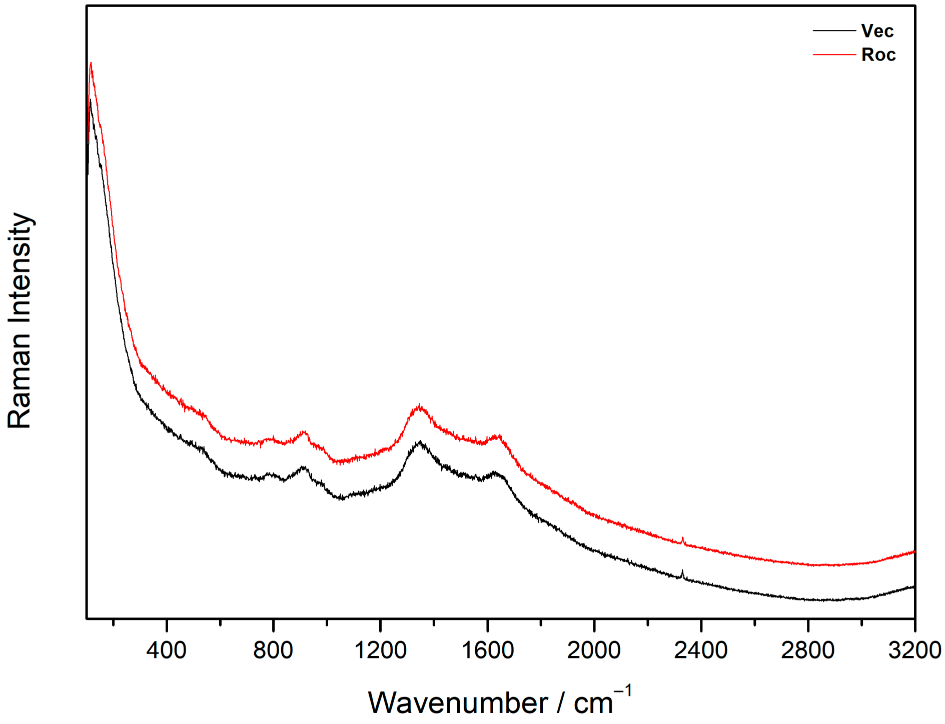

Figure S2. Raman spectra of the drug stock solutions (1×10^{-2} M).

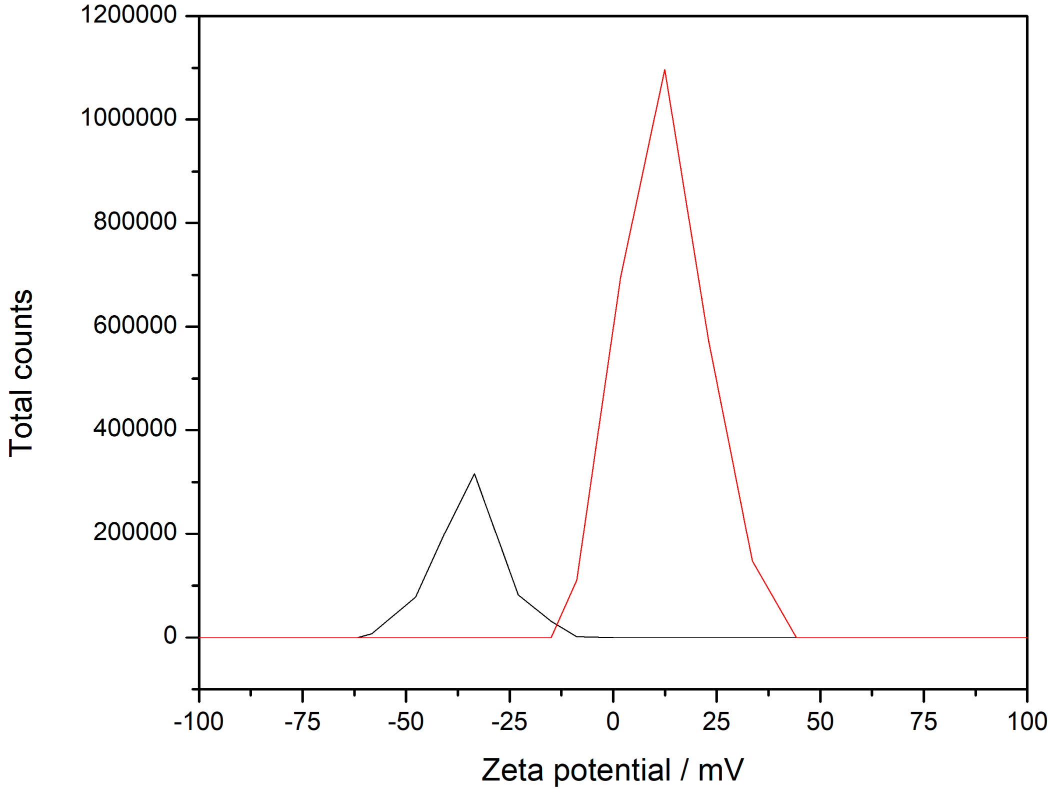

Figure S3. Zeta potential distribution for the silver colloid (black line) and the colloid with Vecuronium (red line).

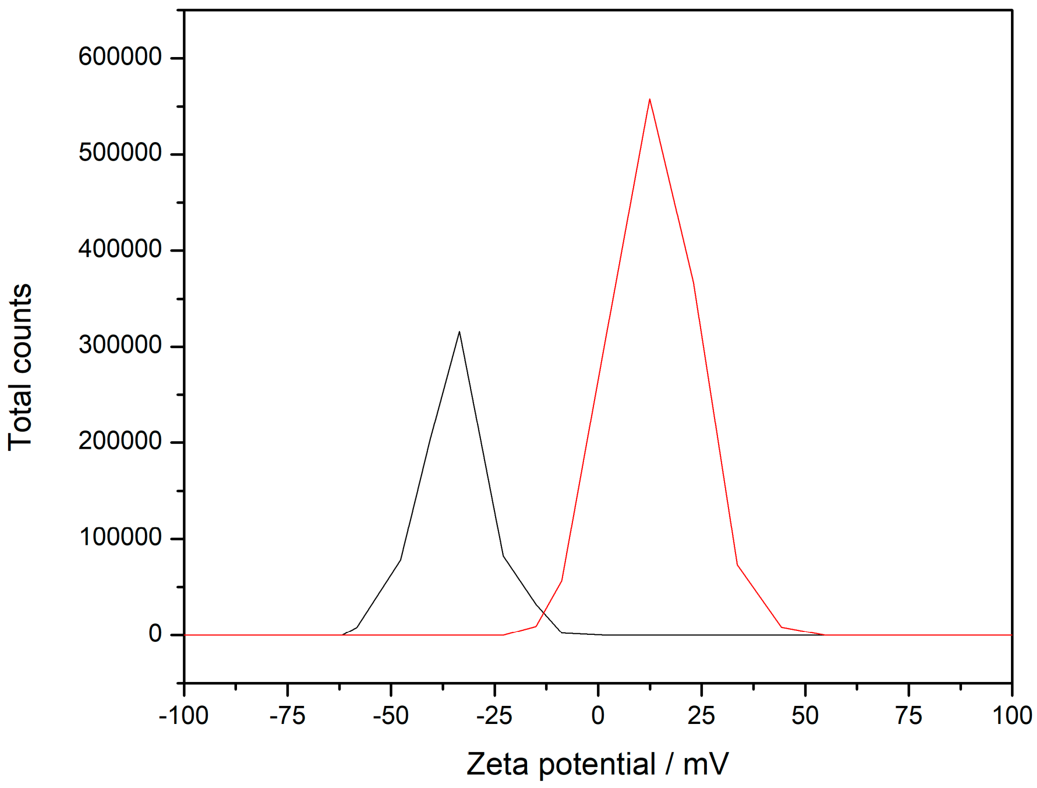

Figure S4. Zeta potential distribution for the silver colloid (black line) and the colloid with Rocuronium (red line).

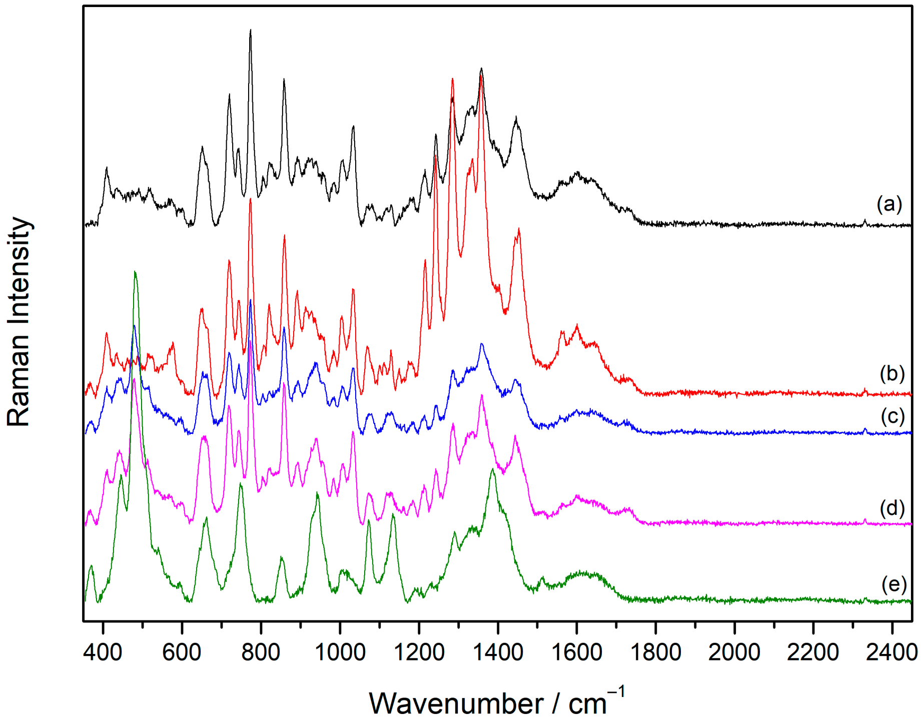

Figure S5. The SERS spectra of **SDX**/**Vec** mixtures (c(**SDX**) = c(**Vec**) = 1×10^{-5} M) differing in the order of addition of components according to methods A, B and C, and recorded immediately after the last component was added. The SERS spectra: (a) c(**Vec**) = 1×10^{-5} M, (b) **SDX**/**Vec** mixture prepared by method A, (c) **SDX**/**Vec** mixture prepared by method B, (d) **SDX**/**Vec** mixture prepared by method C, and (e) c(**SDX**) = 1×10^{-5} mol/L. The spectra are displaced for visual clarity.

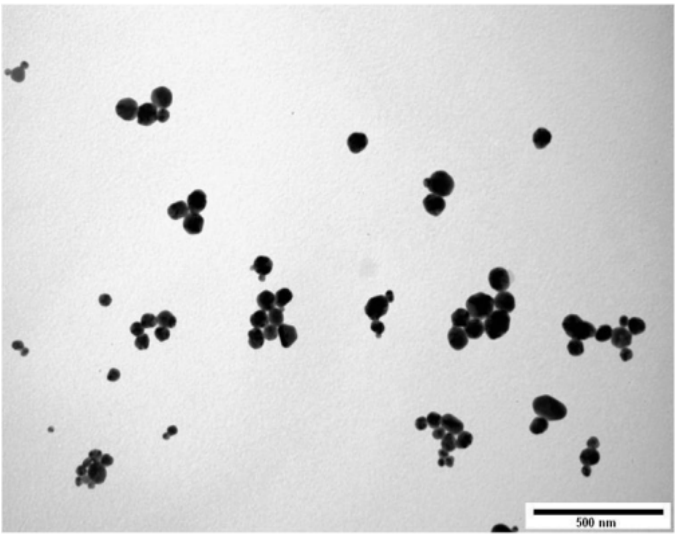

Figure S6. TEM micrograph of colloidal nanoparticles.
